# Quantifying Neighborhood-Scale Spatial Variations of Ozone at Open Space and Urban Sites in Boulder, Colorado Using Low-Cost Sensor Technology

**DOI:** 10.3390/s17092072

**Published:** 2017-09-10

**Authors:** Lucy Cheadle, Lauren Deanes, Kira Sadighi, Joanna Gordon Casey, Ashley Collier-Oxandale, Michael Hannigan

**Affiliations:** 1 Department of Mechanical Engineering, University of Colorado Boulder, Boulder 80309, CO, USA; Kira.Sadighi@Colorado.edu (K.S.); Joanna.Casey@Colorado.edu (J.G.C.); Ashley.Collier@Colorado.edu (A.C.-O.); Michael.Hannigan@Colorado.edu (M.H.); 2 SOARS Program, UCAR, Boulder 80301, CO, USA; Lxd5122@psu.edu

**Keywords:** ozone, spatial variability, air pollution, exposure science, low-cost sensors

## Abstract

Recent advances in air pollution sensors have led to a new wave of low-cost measurement systems that can be deployed in dense networks to capture small-scale spatio-temporal variations in ozone, a pollutant known to cause negative human health impacts. This study deployed a network of seven low-cost ozone metal oxide sensor systems (UPods) in both an open space and an urban location in Boulder, Colorado during June and July of 2015, to quantify ozone variations on spatial scales ranging from 12 m between UPods to 6.7 km between open space and urban measurement sites with a measurement uncertainty of ~5 ppb. The results showed spatial variability of ozone at both deployment sites, with the largest differences between UPod measurements occurring during the afternoons. The peak median hourly difference between UPods was 6 ppb at 1:00 p.m. at the open space site, and 11 ppb at 4:00 p.m. at the urban site. Overall, the urban ozone measurements were higher than in the open space measurements. This study evaluates the effectiveness of using low-cost sensors to capture microscale spatial and temporal variation of ozone; additionally, it highlights the importance of field calibrations and measurement uncertainty quantification when deploying low-cost sensors.

## 1. Introduction

Surface level ozone is well established as harmful to human health, causing impaired lung function in both healthy and sensitive populations [1,2,3]. The EPA regulates ground-level ozone via the National Ambient Air Quality Standards (NAAQS) (80 FR 65291) by specifying that the three-year average of the fourth-highest daily maximum 8-h average concentration of ozone in ambient air cannot exceed 70 ppb, lowered from the 75 ppb standard in place during this study [4]. The Denver Metropolitan and Northern Front Range Region of Colorado has been designated by the EPA as a “nonattainment” area for ozone since 2007 [4]. Colorado is a unique setting for surface ozone formation as urban emissions from highly populated areas and point sources such as power plants mix with emissions from nearby and rural oil and natural gas activities in the Wattenberg Gas Field.

Criteria pollutants, including ozone, are continuously monitored in the U.S. at standard regulatory air quality monitoring (AQM) stations. These measurements are of high quality but are sparsely distributed and unsuited for providing high spatial resolution variations in concentration [5,6,7]. Recent advances in air pollution sensors and embedded systems have led to a new wave of low-cost measurement systems that can be deployed in dense networks to capture small-scale spatio-temporal variations in air pollutants [5,7,8,9,10,11]. Low-cost sensor networks have the potential to improve spatio-temporal resolution of pollutant data collection because they have lower capital and operating costs than conventional fixed-site monitors [11,12]. Understanding spatial variations of pollutants in ambient air could help improve mitigation strategies and enhance personal exposure monitoring on a neighborhood scale [10,12].

Multiple studies have evaluated the feasibility of deploying sensor networks to measure ozone in urban settings, and to measure spatial variability of air pollutants; different field calibration methods have been tested in an effort to improve data quality [5,6,7,8,11,13]. A few of these studies employ the assumption that all sensors within a certain proximity of each other measure the same value as reference instruments from 1:00 to 4:00 a.m., when there is no photochemical production of ozone [5,7]. Even during these hours, there could be point sources of NO initiating ozone depletion or variances in ozone deposition rates that are not necessarily constant over the spatial scale being considered. Another limitation in previous studies is the lower time resolution of reference data (30 min to 1 h). Higher time resolution measurements could potentially result in improved field calibrations and data quality, as well as decreased uncertainty due to the inclusion of a broader range of variables such a temperature and humidity. The spatial scales studied in previous work ranged from 150 m to 150 km between measurement locations, but there is limited research to date documented in the literature evaluating the spatial variability of ozone on scales less than 150 m.

The study described here contains three spatio-temporal process scales as defined by Diem [14]. We analyzed ozone variability on a microscale (up to tens of meters, several hours), and on a mesoscale (tens of kilometers, several hours to several days). We logged the signal from each ozone sensor every 15 s. The reference data used for field calibrations were minute averages (open space site) and 5-min averages (urban site). Our study compares the small spatial variations in ozone at the open space site versus the urban site and examines the overall differences between the two sites. 

## 2. Materials and Methods

In order to demonstrate the utility of low-cost sensor systems for assessing open space and urban microscale spatial variability, we deployed a fleet of seven metal oxide ozone sensor systems in the Boulder, Colorado area. The sensor systems used for this study were based on the UPod platform [15]. This study, which took place during June and July of 2015, was composed of two main parts: (1) the deployment of UPods at an urban and open space site to measure the spatial variability of ozone on small scales, and (2) the collocation of UPods with reference stations to generate calibration models. The ozone sensors were quantified using a field calibration technique, described in detail by Piedrahita and colleagues [16].

### 2.1. UPod Platform

The ozone sensors used were the MiCS-2611 heated metal oxide sensors, manufactured by e2v, now SGX Sensortech [17]. Heated metal oxide sensors used to measure ozone sometimes display cross sensitivity to H_2_S, a reducing gas, but this is not a common occurrence in most environments [18]. The sensors were mounted on the open-source UPod platform which can house a number of low-cost gas sensors [19]. In this study, each UPod unit contained ozone, temperature, and relative humidity (RH) sensors. Temperature and RH were measured using the RHT03 sensor that is manufactured by MaxDetect (Shenzhen, China) [20]. Data from each sensor was written to an onboard microSD card every 15 s, then minute averages were calculated in post processing. The UPods were designed to run on a 12 V power supply, and in this study six out of the seven UPods were powered using grid electricity, employing AC/DC converters and extension cords. During the deployment, one UPod (SBC4) was located beyond reach of an extension cord and was powered using a 12 V deep cycle marine battery and solar panel configuration. The UPod circuit boards were mounted in enclosures that included an electric fan to augment their air exchange rate.

### 2.2. Deployments

**Figure 1:**
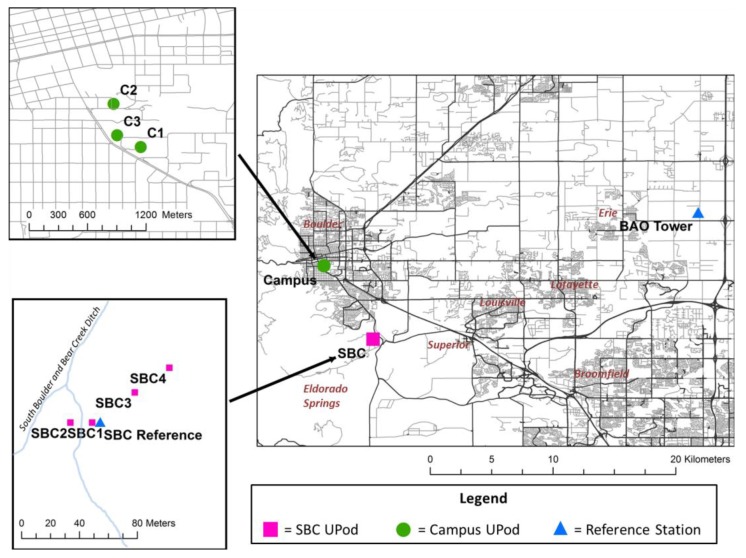
Maps of study area including overall collocation and deployment sites, campus UPod locations, and South Boulder Creek (SBC) UPod locations.

The deployments took place at an urban site and an open space site. The urban site was on the University of Colorado Boulder campus (student body of 32,775), located in south Boulder, Colorado, (population of 120,723) 40 km northwest of Denver. The major road nearest to the UPods was Broadway, which had a 2015 Annual Average Daily Traffic Count (AADT) of 30,000 [21]. The open space site was located at the South Boulder Creek (SBC) regulatory ozone monitoring station, stationed south of Boulder in a highly vegetated and sparsely populated area (latitude 39.9572, longitude −105.0004, 1671 meters above sea level (masl). The SBC station was classified by the Colorado Department of Public Health and Environment (CDPHE) as a highest-concentration oriented urban monitor, but for purposes of this study was considered an open space area with fewer buildings and nearby roads than the campus site. The SBC site was 0.4 km west-southwest from Highway 93, which had an AADT of 18,000 in 2015 and was less trafficked than the campus site [21]. Three UPods (C1, C2, and C3), were deployed on the CU campus and four UPods (SBC1, SBC2, SBC3, and SBC4), were deployed in the SBC area (6.5 km southeast of campus). A map of the two study areas and reference stations is shown in Figure 1.

The deployments spanned 25 June to 14 July for the campus UPods and 30 June to 12 July for the SBC UPods. For all data analysis, only the time period when all UPods were sampling, 30 June to 12 July, was included. The deployment locations of all UPods are summarized in detail in Table 1. Altitudes of campus UPod measurement sites ranged from 1645–1657 masl and all SBC UPods were at ~1671 masl. All UPods were mounted onto tripods, sampling air 1.5 m above the ground. The campus UPods’ tripods were located on two rooftops and a balcony and were all ~1 m or greater from the nearest wall, while the SBC UPods’ tripods were on ground level. Distances between UPods ranged from 221–491 m at the urban site, 12–66 m at the open space site, and approximately 6.2–6.7 km between the urban and open space UPods.

### 2.3. Calibration

The calibration portion of the study consisted of a collocation period when UPods sampled similar air as a reference monitor in order to generate a calibration for the sensors, often termed a field normalization. Three of the UPods, C1, C2, and C3, were collocated with the Boulder Atmospheric Observatory (BAO) tower surface ozone monitor from 15 June to 18 June, and four of the UPods, SBC1, SBC2, SBC3, and SBC4, were collocated at the SBC reference station 15–25 June.

The BAO Tower was run by the National Oceanic and Atmospheric Administration (NOAA), which operated a continuous UV absorption Thermo-Scientific Ozone Monitor (49c, 3711) with an inlet that was 6 m above the ground [22]. Ozone observations from BAO Tower have undergone thorough evaluation and quality control following calibration procedures available through NOAA [23]. The reference data was available in 5-min averaged form. As such, that was the temporal resolution used for the calibration of the ozone sensors in the campus UPods. The SBC reference site (AQS Site # 08-013-7005) operated a Teledyne Model 400E Photometric Ozone analyzer maintained by CDPHE and calibrated in accordance with U.S. EPA protocols [24]. This data was provided with minute resolution and was used at that resolution for the calibration of the SBC UPods. Higher time resolution measurements could potentially result in improved field calibrations and data quality, and decreased uncertainty due to the inclusion of a broader range of variables such as temperature and humidity. Therefore, the calibration at SBC might be more accurate than that at BAO due to the higher time resolution of reference data (1 min averages versus 5 min averages, respectively). During collocations, all UPods were placed on tripods ~3–4 m below the reference instrument inlets.

Ozone sensors in each UPod were calibrated using the reference data from the site where they were collocated with reference instruments. Multiple linear regression was used to generate a model to convert the raw sensor signal into a concentration (in ppb). We found the sensor resistance as a function of the logged voltages then normalized the sensor resistance, *R_s_*, by the sensor signal in clean air at 298 K, *R_o_*. The regression Equations (1)–(4) relate *R_s_*/*R_o_* to the reference instrument concentration (C), temperature (T), and absolute humidity (H); T and RH terms were included to account for the cross-sensitivities of heated metal oxide sensors to those parameters [16,19]. RH was converted to H using methods described by Murphy and Kook and assuming constant atmospheric pressure of 82 kPa [25]. The coefficients *p*_1_, *p*_2_, *p*_3_, *p*_4_, and *p*_5_ were computed each time a model was generated.

*R_s_/R_o_* = *p*_1_ + *p*_2_C (1)

*R_s_/R_o_* = *p*_1_ + *p*_2_C + *p*_3_T (2)

*R_s_/R_o_* = *p*_1_ + *p*_2_C + *p*_3_T + *p*_4_H (3)

*R_s_/R_o_* = *p*_1_ + *p*_2_*C* + *p*_3_T + *p*_4_H + *p*_5_CT (4)

The fit of the calibration models to the reference data was evaluated using the coefficient of determination (*R*^2^), the root-mean-square error (RMSE), and the distribution of the fit residuals with concentration, humidity, and temperature. Residuals were calculated by subtracting the calibrated UPod data from the reference data during the collocation time period. Regression analysis is based on the assumption of normally distributed residuals so calibration equations that generated approximately normally distributed residuals were deemed better fits than those that did not.

At the SBC site, part of the reference-UPod collocation data was used to generate calibration models for ozone sensors in each UPod, and the rest of the reference-UPod collocation data was used toward validation of the calibration models. As such, we used the second half of the collocation period at SBC, 20 June to 25 June, to generate a calibration model for each UPod, and that model was then applied to the first half, 15 June to 19 June. This reverse temporal order was chosen to minimize the impact of sensor drift over time; the time period used to generate a calibration model was in between the time periods it was applied to (validation and deployment). The fit statistics for the model applied to the first half represents the validation. A similar validation procedure was not completed at the BAO site because a wind storm limited our data collection during that time period.

We generated a calibration dataset for each collocation period by applying the calibration models to the UPod data during collocation periods at both SBC (20 June to 25 June) and BAO Tower (15 June to 18 June). The two calibration datasets represent the best possible agreement of UPod ozone measurements; differences in UPod measurements during the deployment that are less than the calibration differences would be too small to resolve.

## 3. Results and Discussion

### 3.1. Calibration Results

Raw data from each of the 7 UPods was fit to Equations (1)–(4) and the *R*^2^ and RMSE were calculated for each model. An example of model selection process for UPod SBC1 is summarized in Supplementary Materials (Table S1); this process was repeated for each of the 7 UPods. Residuals were plotted for each UPod and model to check for the assumption of normality. The residuals for the calibration of SBC1 using Equation (4) are shown as an example in Supplementary Materials (Figure S1). Residuals were plotted against concentration, humidity, and temperature to verify that sensor error was not biased based on environmental conditions. The parameter space encompassed by the calibration models was compared to the data validation and deployment periods to evaluate the extent of model extrapolation (see Supplementary Materials Figure S2). Using this model selection process, Equation (4) was chosen for each of the UPods, and the fit statistics are shown in Table 2. The UPods all had similar *R*^2^ (between 0.91 and 0.97) as well as similar RMSE (between 2.4 and 5 ppb, with an average of 3.2 ppb). The calibration RMSE represents the smallest potential uncertainty in the sensor measurements given that the reference instrument data are assumed to be the truth, i.e., contain no uncertainty. Contributions of terms in Equation (4) to *R_s_/R_o_* for SBC UPods during the data validation period are summarized in Supplementary Materials (Figure S3). *R_s_/R_o_* had a higher *R*^2^ with concentration than with temperature or humidity, but temperature did play a larger role at higher concentrations. Correlations between *R_s_/R_o_* and temperature and ozone concentration were higher for SBC3 than for SBC1 or SBC2, due to the sensor-to-sensor variation in calibration models. For SBC3, the CT term in Equation (4) likely did not contribute as much to *R_s_/R_o_* as it did for SBC1 and SBC2. This demonstrates the importance of calibrating each sensor individually to accurately capture the influence of the different terms in each calibration model.

### 3.2. Validation and Uncertainty Estimation

**Figure 2:**
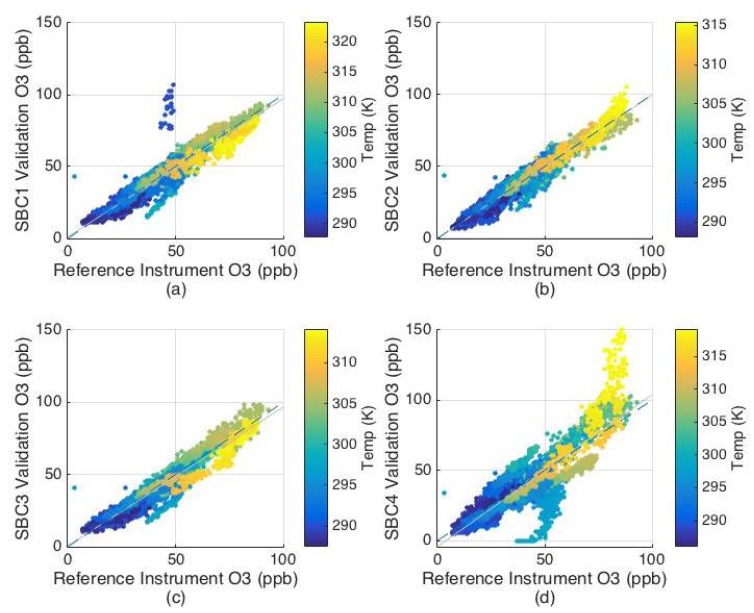
Scatterplots of sensor data versus reference data for UPod SBC1 (**a**); SBC2 (**b**); SBC3 (**c**); and SBC4 (**d**) during the validation collocation.

The performance of the SBC calibration was evaluated using the validation dataset. The calibrated collocation data during the validation period for the four UPods (SBC1, SBC2, SBC3, and SBC4) were compared to the reference data using the *R*^2^ and RMSE and the results are shown in Table 2. Scatterplots of the UPod data versus the reference data are shown in Figure 2. SBC1, SBC2, and SBC3, all showed similar performance with an average RMSE of 5 ppb. The cluster of outliers at lower temperatures in the SBC1 plot (Figure 2a) is the result of a spike in the data on 16 June (see Supplementary Materials, Figure S4). Given that instantaneous spikes occurred simultaneously in the data for ozone, T, and RH, as well as for other gas sensors that were present in UPods during the study but not included in this analysis (CO_2_ and VOCs), it is likely that this spike in the June 16 data was caused be a power issue affecting the sensors. SBC4 did not perform as well as the other SBC UPods with an *R*^2^ of 0.73 and an RMSE of 12 ppb. Additional error analysis for SBC1, SBC2, and SBC3, was completed to evaluate the RMSE for higher ozone concentrations, since we are typically interested in higher ozone levels and their associated uncertainty. For concentrations >60 ppb, the average RMSE for the three SBC pods during validation was 5.7 ppb, verifying that the calibration models measured similar error with the reference instrument during high ozone measurements.

The poor performance of sensor SBC4 was investigated further; see Supplementary Materials (Figure S4). The largest discrepancy between SBC4 and the reference data occurred during the middle of the day on 18 June. SBC4 shifted abruptly from overestimating to underestimating, and this event was not observed with the other SBC UPods. A potential cause of this event could have been loss of power to the UPod, given the solar configuration of SBC4, but due to the instantaneous nature of the shift it is unlikely to have been produced by a power disruption and so remains unexplained. SBC4 also appeared to over and under predict ozone concentrations relative to other pods (see Figure S4). Accordingly, SBC4 data was not included in the spatial variability analysis at SBC.

Sensor baseline drift over time is a common issue for heated metal oxide sensors [16,19]. We tested a calibration model with an additional term to account for temporal drift and found that the model performance did not improve when applied to the validation data. For example, SBC2 had the same *R*^2^ with reference data when the temporal drift term was included in the calibration model, however, the RMSE increased by 0.1 ppb compared to the Equation (4) model. Future studies with longer durations of multiple weeks to months may benefit from incorporating sensor baseline drift into their calibration models, but for the relatively short-term nature of this study, adding a term for temporal drift did not improve the calibration model performance.

To frame the discussion of spatial variability, the average uncertainty of 5 ppb from the validation dataset was employed as this represents a more realistic estimate of best possible sensor agreement during the deployment. The 5 ppb average uncertainty was applied for both the SBC and campus UPods since a similar validation procedure was not completed for the BAO collocation. The issue with SBC4 is motivation to improve the hardware and software of low-cost tools like the UPod to minimize data losses and discontinuity in data as recorded by SBC4. Until low-cost sensing tools and quantification models become more robust, added redundancy and focus on validation of quantification methods are necessary.

### 3.3. Spatiotemporal Variability

**Figure 3:**
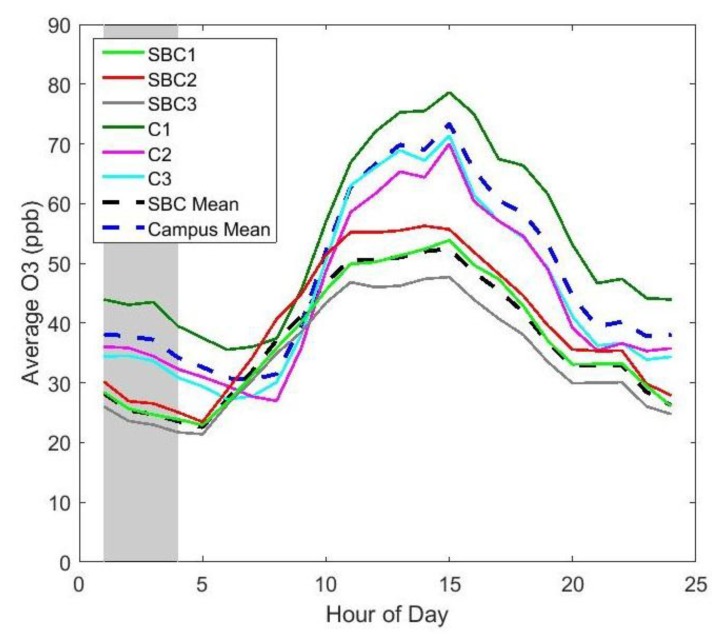
Average ozone measurements at each hour of the day during the deployment period for all UPods and averaged campus and SBC sites. Hours 1–4 are highlighted in gray.

Figure 3 shows the average diurnal ozone concentration measured by each UPod for the duration of the deployment. UPods at the two sites measured concentrations that exhibited inter- and intra-site variability. The SBC UPods measured a slightly different diurnal shape from the campus UPods with ozone growth beginning earlier in the morning. Differences between UPods were the largest in magnitude in the afternoon and C1 recorded the highest average maximum concentrations of the campus UPods with SBC2 the highest of the SBC UPods. Furthermore, between the highlighted hours of 1:00 and 4:00 a.m. (all times in this study in local time), there were differences in the UPod measurements, indicating that previous studies’ assumptions of homogeneous ozone concentrations during that time period may not be valid, and could introduce error to the results. The magnitude of this potential error would likely vary based on the location of the study; future studies are recommended to quantify the potential error introduced into a deployment when utilizing the homogeneous ozone concentration assumption to calibrate sensors. This error could be compared between multiple locations to determine for which sites this assumption may be valid, and for which it may introduce significant error. The spatial variation of nighttime ozone demonstrated in Figure 3 could also be influenced by nighttime chemistry of ozone reacting with NO*x*, as well as by varying deposition rates between sites. 

The concentrations measured by the campus UPods were on average greater than the SBC UPods, which differed from a previous study in 2008 that measured higher annual average concentrations at SBC than in Boulder at a site slightly northeast of campus [26]. This difference could be due to a number of factors, one being that the previous study had a much longer duration, and the time period captured in this deployment may not be representative of overall trends at the two sites. In addition, the SBC and campus UPods were collocated at different locations and using different reference data, which could have impacted the results. Future deployments should include a validation period where all UPods are collocated together for comparison with the same reference data. The difference in ozone between SBC and the campus is important to consider in terms of human exposure in Boulder, and potentially other urban areas of similar size and ozone precursor emissions because people in more populated areas may be exposed to higher ozone levels than those in more open space areas.

The *R*^2^ values that reflect the agreement between UPods were calculated for the deployment data to assess microscale and mesoscale correlation of UPods. Intra-site correlation between UPods was high at both locations with *R*^2^ values ranging from 0.88 to 0.93 for SBC UPods and 0.88 to 0.93 for campus UPods. Correlation between the two sites was lower with the *R*^2^ values for SBC and campus UPods between 0.54 and 0.63. In this study, correlation on the microscale was higher than on the mesoscale, however correlation is not the only important indicator of spatial variability. Figure 3 demonstrates that even on the microscale where UPods were highly correlated there were still measured differences in ozone. These results identify the need to evaluate both absolute differences, and correlation between sites on the microscale. However, on the mesoscale, some differences may be observed by considering correlation alone.

**Figure 4:**
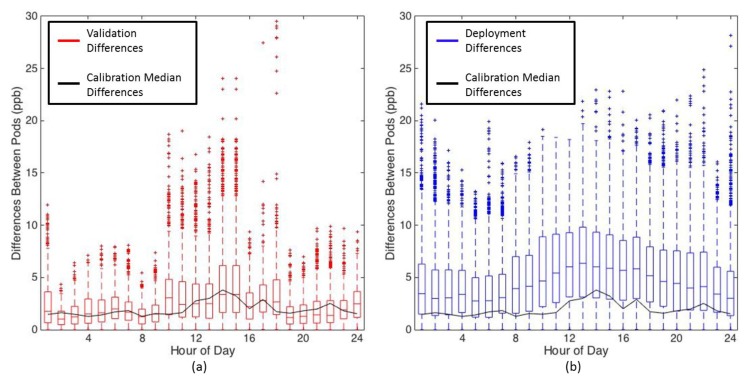
Differences between all SBC UPods binned by hour, with differences displayed as absolute ppb. (**a**) includes the boxplot of the differences during the data validation period (red outlined boxes), and the median differences of the calibration data (solid black line). (**b**) includes the boxplot of the differences during the deployment (blue outlined boxes), and the median differences of the calibration data (solid black line). Whisker lines (red and blue) encompass 1.5 times the interquartile range (IQR), and outliers (red and blue crosses) indicate data points lying outside 1.5 times the IQR of the validation and deployment data, respectively.

Differences in the hourly ozone observed at each site can be seen in Figure 3, however it is not clear from this plot if the intra-site ozone differences were due to measurement error or if real spatial variability was being observed on micro intra-open space and intra-urban scales. Figure 4 shows the differences between all SBC UPods, as boxplots binned by hour of the day. The boxplots include the validation data, the deployment data, and the median differences of the calibration data, showing that the UPods measured smaller differences during the collocation period than during the deployment period. The red boxplots of the validation dataset represent the expected intrinsic disagreement among the UPods that can be attributed to uncertainty. The black median line represents disagreement among the UPods during the calibration model generation period, which matches nicely with the validation dataset disagreement. The validation and calibration differences were greatest in magnitude in the afternoon when ozone was the highest, indicating that sensor uncertainty is larger during periods of higher ozone. The upper 75% of deployment differences lie well above the validation differences, especially between the hours of 10:00 a.m. and 8:00 p.m., demonstrating that some differences observed at the SBC sites were greater than their uncertainty, and spatial variability on a micro intra-open space scale was observed. Since SBC4 was omitted from this analysis, the total distances between the SBC UPods ranged from 12 to 41 m. 

**Figure 5:**
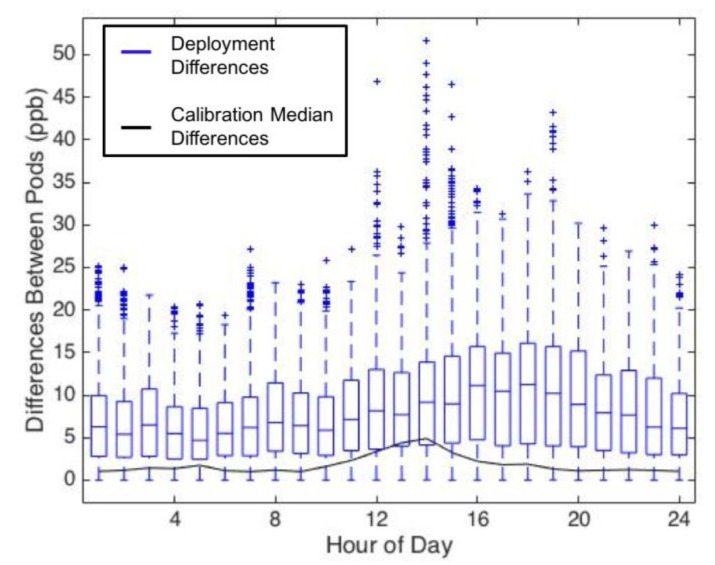
Differences between all campus UPods binned by hour, with differences displayed as absolute ppb. The graphic includes the boxplot of the differences during the deployment (blue outlined boxes), and the median differences of the calibration data (solid black line). Whisker lines (blue) encompass 1.5 times the IQR, and outliers (blue crosses) indicate data points lying outside 1.5 times the IQR of the deployment data.

Figure 5 shows the hourly difference boxplot for the campus UPods, although without the validation dataset since that was only collected for the SBC UPods. The magnitudes of the differences between the campus UPods were greater than the SBC UPods, with a maximum median difference of 11 ppb for the campus UPods, compared to 6 ppb for the SBC UPods. There are multiple factors that could have led to larger differences between campus UPods than SBC UPods. The campus UPods were in a more urban environment with potentially more point source emissions, they had larger separation distances than the SBC UPods, and they differed slightly in altitude (see Table 1). Additionally, they may have experienced more differences in shade and temperature due to the nearby buildings. For all hours of the day, the median differences between campus UPods were well above the median differences measured during the calibration, indicating that the spatial variability is greater than the uncertainty of the differences, even during the higher uncertainty period of the afternoon when the ozone levels are the highest. Interestingly, from 1:00 to 4:00 a.m., the median differences were still above 5 ppb, suggesting small spatial scale variability of ozone in an urban setting even during that time period.

In both Figure 4 and Figure 5, there is a strong diurnal trend where the differences between UPods were the greatest in the afternoons when the ozone levels were the highest (see Figure 3 for average ozone levels by hour). This shows that there was greater spatial variability of ozone during the times of peak ozone production. Overnight and in the early morning, there was less spatial variability. It is interesting to note that the maximum differences between UPods occurred at different times between the SBC and campus sites. Within the SBC UPods, the maximum difference occurred during hour 13 (noon to 1:00 p.m.) with a median difference of 6 ppb. On campus, however, the maximum difference between UPods was during hour 16 (3:00 p.m. to 4:00 p.m.) with a median difference of 11 ppb. This demonstrates that the spatial variability observed is not the same between the two sites, and the microscale ozone variability may be impacted by different sources in an open space versus an urban setting.

### 3.4. Impact of Time Averaging

The time-averaging results are important when considering ozone spatial variability in a regulatory context, versus a human health context. Exceedances of the NAAQS for ozone are evaluated using 8-h averaged data, and this study found that spatial differences were smaller over the regulatory timescale. Ozone exposure can impact human health on one-hour timescales and the large differences observed in the hour data could lead to different exposure potentials on small spatial scales [27,28]. Spatial variability of ozone on the small scale presented in this study is dependent on the time-averaging of measurements as shown in Table 3, which contains the deployment median and 95th percentile ozone values for each of the UPods over three time-averaging scales of minute, hour, and 8-h (rolling). For both the SBC and campus deployments, the median ozone values for each UPod were similar across all time-averaging scales. The spatial differences between UPods’ 95th percentile ozone values were smaller in magnitude in the 8-h averaged 95th percentile data than in the minute or hour averaged data. Spatial variability is observed between UPods over all time-averaging scales, but is reduced for larger time-averaging.

**Figure 6:**
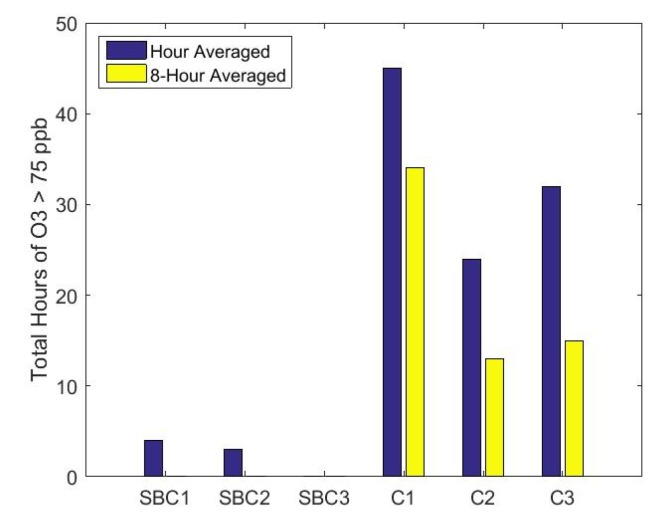
Total hours of ozone measurements greater than 75 ppb during deployment for 1-h and 8-h averaged data.

The spatial differences in high ozone measurements are investigated further in Figure 6 with a comparison of the total time concentrations, which were greater than 75 ppb for each UPod during the deployment. There were differences between campus UPods with C1 measuring more hours above 75 ppb than C2 and C3 over both time-averaging scales. The magnitudes of the differences between campus UPods were reduced for 8-h time-averaging compared to hour, especially when comparing C2 and C3. Differences between SBC UPods were smaller in magnitude but still present, with SBC1 and SBC2 having similar times above 75 ppb for hour averaged data, and no time above 75 ppb for 8-h data. SBC3 had no measurements above 75 ppb on either time-averaging scale. The spatial differences observed at SBC for hour data are not present at the 8-h level when all UPods were below 75 ppb.

## 4. Conclusions

Overall, the results from this study indicate that there is both intra-open space and intra-urban spatial variability of ozone on very small spatial scales ranging from 12 m (smallest SBC difference) to 6.7 km (largest difference between all UPods). Low-cost sensor systems such as the UPod used in this study are able to quantify these small variations and provide accurate results within a given uncertainty range. Measurements using low-cost sensors could be useful for future human health exposure studies to help quantify spatial variability of ozone in neighborhood settings. This study demonstrates the importance of performing collocations in the field to generate accurate calibrations; validation datasets are also important in evaluating calibration performance, and obtaining accurate uncertainty estimates that are needed to frame the deployment results.

## Figures and Tables

**Table 1:** Summary of Measurement Sites.

Ozone Instrument Name	Instrument Type	Latitude	Longitude	Location Altitude (m above sea level)	Location Description	Inlet Height (m above ground)	Collocation/Deployment
Boulder Atmospheric Observatory (BAO) Tower	UV Absorption Analyzer	40.0500	−105.0004	1584	Tall tower operated by the National Oceanic and Atmopsheric Administration (NOAA)	6	Collocation
SBC-Ref	Photometric Ozone Analyzer	39.9572	−105.2385	1671	Colorado Department of Public Health and Environment (CDPHE) monitoring site	4.3	Collocation
C1	Metal Oxide Sensor	40.0069	−105.2720	1667 (including 15 m from ground to rooftop)	University Memorial Center building—rooftop	1.5	Collocation (at BAO) and Deployment
C2	Metal Oxide Sensor	40.0109	−105.2745	1655 (including 10 m from ground to rooftop)	Continuing Education building—western rooftop	1.5	Collocation (at BAO) and Deployment
C3	Metal Oxide Sensor	40.0080	−105.2742	1666 (including 9 m from ground to balcony	Geography building—south balcony	1.5	Collocation (at BAO) and Deployment
SBC1	Metal Oxide Sensor	39.9572	−105.2386	1671	SBC—nearest to reference monitor	1.5	Collocation (at SBC) and Deployment
SBC2	Metal Oxide Sensor	39.9572	−105.2387	1671	SBC – nearest to trees and more dense foliage	1.5	Collocation (at SBC) and Deployment
SBC3	Metal Oxide Sensor	39.9575	−105.2381	1671	SBC—nearest to road	1.5	Collocation (at SBC) and Deployment
SBC4	Metal Oxide Sensor	39.9574	−105.2383	1671	SBC	1.5	Collocation (at SBC) and Deployment

**Table 2:** Sensor Calibration and Validation Collocation Results.

Segment of Collocation	Pod ID	*R*^2^ with Reference Instrument	RMSE with Reference Instrument (ppb)
Calibration Generation Period	SBC1	0.95	3.2
	SBC2	0.95	3.2
	SBC3	0.97	3.0
	SBC4	0.93	5.0
	C1	0.91	2.9
	C2	0.91	2.4
	C3	0.92	2.5
Validation Data Period	SBC1	0.90	5.9
	SBC2	0.95	4.3
	SBC3	0.92	5.3
	SBC4	0.73	12.3

**Table 3:** Median and 95th Percentile Ozone for Minute, Hour, and 8-H Averaged Deployment Data.

Pod ID	Median Ozone (ppb)			95th Percentile Ozone (ppb)		
	Minute	Hour	8-h	Minute	Hour	8-h
SBC1	35	36	35	65	65	58
SBC2	40	39	39	66	66	59
SBC3	33	33	32	58	57	53
C1	51	51	50	93	93	83
C2	39	39	40	83	83	75
C3	39	39	39	87	87	76

## Supplementary Materials

The following are available online at http://www.mdpi.com/1424-8220/17/9/2072/s1, Table S1: Model Fit Testing Results for UPod SBC1, Figure S1: Residuals for SBC1 collocation data calibrated using Equation (4), Figure S2: Normalized histograms showing the parameter space of ozone, temperature, and humidity during the various data collection periods (calibration generation, data validation, and deployment) for both SBC and Campus UPods. The SBC calibration period encompassed the parameter space of the other periods well, while the campus deployment measured slightly higher ozone, temperature, and relative humidity than the calibration generation period. We can be more confident in our SBC calibration models than our campus calibration models because we are not extrapolating as much into different environments than we measured during the calibration generation period, Figure S3: Scatterplots of *R_s_/R*_o_ vs. temperature, humidity, and ozone for each SBC UPod during the data validation period. The scatterplots demonstrate the correlation between the various terms in the calibration model to the sensor response. *R*^2^ values indicate correlation between *R_s_/R*_o_ and the variable plotted on the x-axis. The sensor signals were more correlated with ozone concentration than with either temperature or humidity for all UPods, Table S2: P-Values for F Test that Calibration Model Coefficient Estimates are Zero at the 5% Significance Level, Figure S4: Time series of ozone measurements by SBC1 (a), SBC2 (b), SBC3 (c), and SBC4 (d) during validation data period with UPod data (red) and reference data (blue) shown in each plot.
