# Supplementary material for: Quantifying Neighborhood-Scale Spatial Variations of Ozone at Open Space and Urban Sites in Boulder, Colorado Using Low-Cost Sensor Technology
Source: Sensors (Basel). 2017 Sep 10;17(9):2072. doi: 10.3390/s17092072 (PMC5620960; doi:10.3390/s17092072)
Supplement: Supplementary file 1 [file sensors-17-02072-s001.pdf]

sensors MDPI

Article

Quantifying Neighborhood-Scale Spatial Variations of Ozone at Open Space and Urban Sites in Boulder, Colorado Using Low-Cost Sensor Technology

Lucy Cheadle [1,*], Lauren Deanes [2], Kira Sadighi [1], Joanna Gordon Casey [1], Ashley Collier-Oxandale [1] and Michael Hannigan [1]

[1] Department of Mechanical Engineering, University of Colorado Boulder, Boulder 80309, CO, USA; Kira.Sadighi@Colorado.edu (K.S.); Joanna.Casey@Colorado.edu (J.G.C.); Ashley.Collier@Colorado.edu (A.C.-O.); Michael.Hannigan@Colorado.edu (M.H.)
[2] SOARS Program, UCAR, Boulder 80301, CO, USA; Lxd5122@psu.edu
* Correspondence: Lucy.Cheadle@Colorado.edu; Tel.: +1-206-271-9224

Table S1. Model Fit Testing Results for UPod SBC1.

Equation #	R^2 of R_s/R_o with Model Predictors	RMSE with Reference Instrument (ppb)
1	0.29	12.0
2	0.67	5.2
3	0.88	3.7
4	0.92	3.2

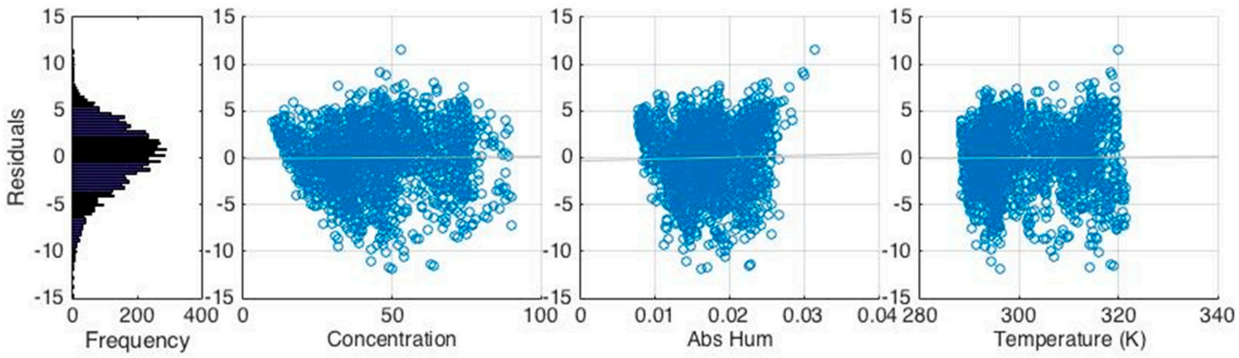

Figure S1. Residuals for SBC1 collocation data calibrated using Equation 4.

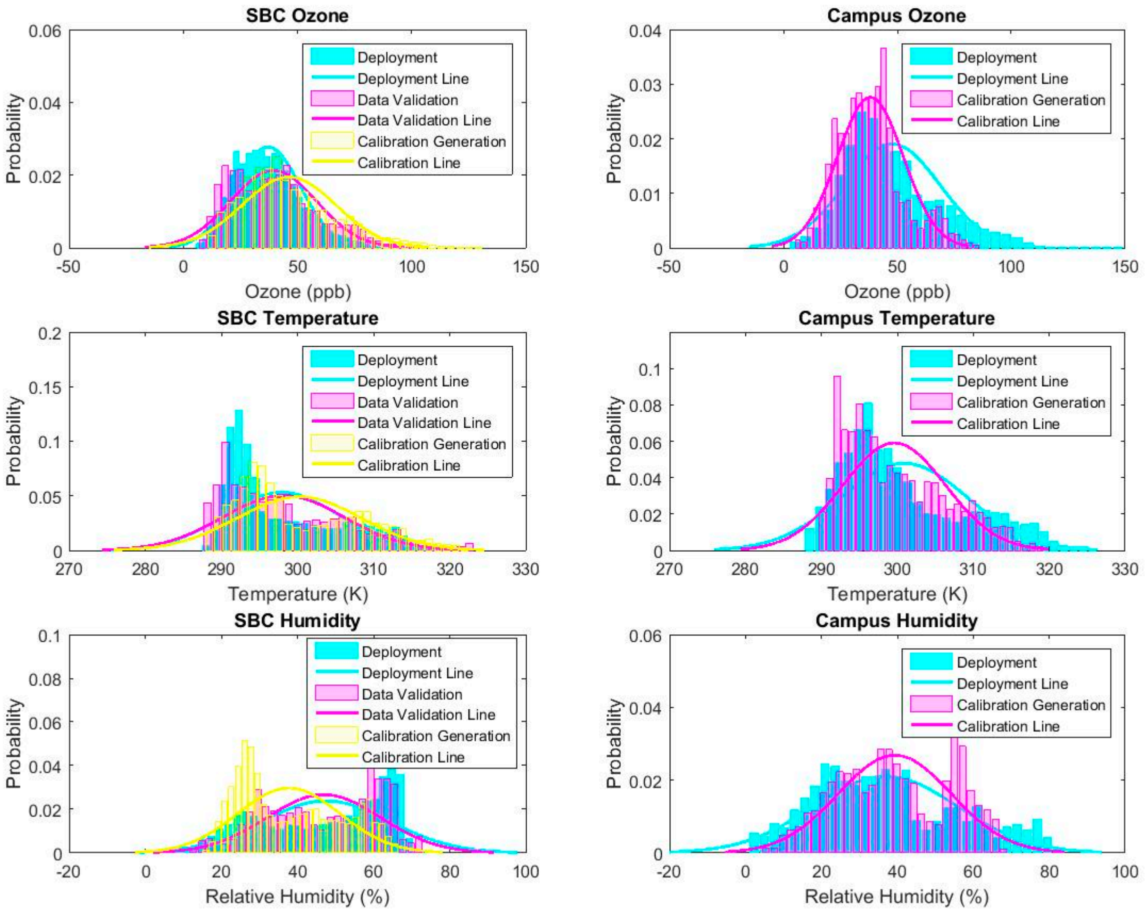

Figure S2. Normalized histograms showing the parameter space of ozone, temperature, and humidity during the various data collection periods (calibration generation, data validation, and deployment) for both SBC and Campus UPods. The SBC calibration period encompassed the parameter space of the other periods well, while the campus deployment measured slightly higher ozone, temperature, and relative humidity than the calibration generation period. We can be more confident in our SBC calibration models than our campus calibration models because we are not extrapolating as much into different environments than we measured during the calibration generation period.

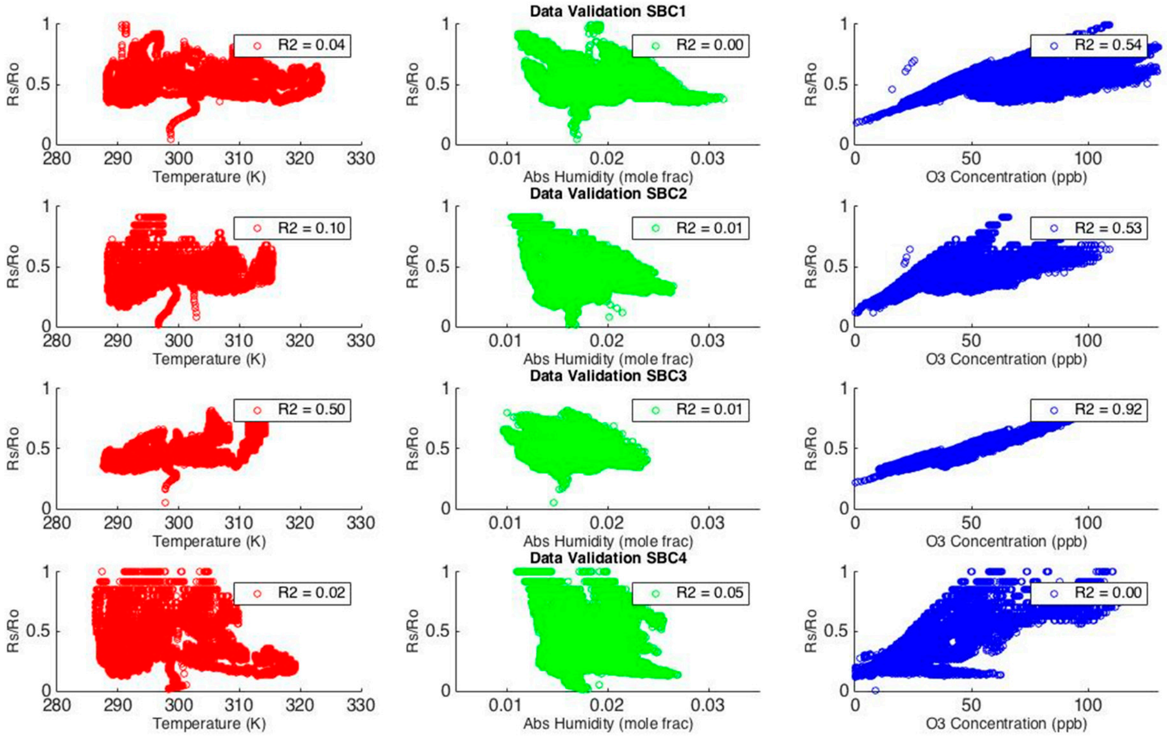

Figure S3. Scatterplots of R_s/R_o vs. temperature, humidity, and ozone for each SBC UPod during the data validation period. The scatterplots demonstrate the correlation between the various terms in the calibration model to the sensor response. R^2 values indicate correlation between R_s/R_o and the variable plotted on the x-axis. The sensor signals were more correlated with ozone concentration than with either temperature or humidity for all UPods.

Table S2. P-Values for F Test that Calibration Model Coefficient Estimates are Zero at the 5% Significance Level.

	Coefficient (and Term Associated with Coefficient)				
**Pod ID**	**p_1 (constant)**	**p_2 (C)**	**p_3 (T)**	**p_4 (H)**	**p_5 (CT)**
SBC1	0.342	0	9.16E-25	0	0
SBC2	1.98E-77	0	4.74E-149	0	0
SBC3	0	1.93E-165	0	0	9.01E-286
SBC4	0.00375	0	1.85E-19	0	0

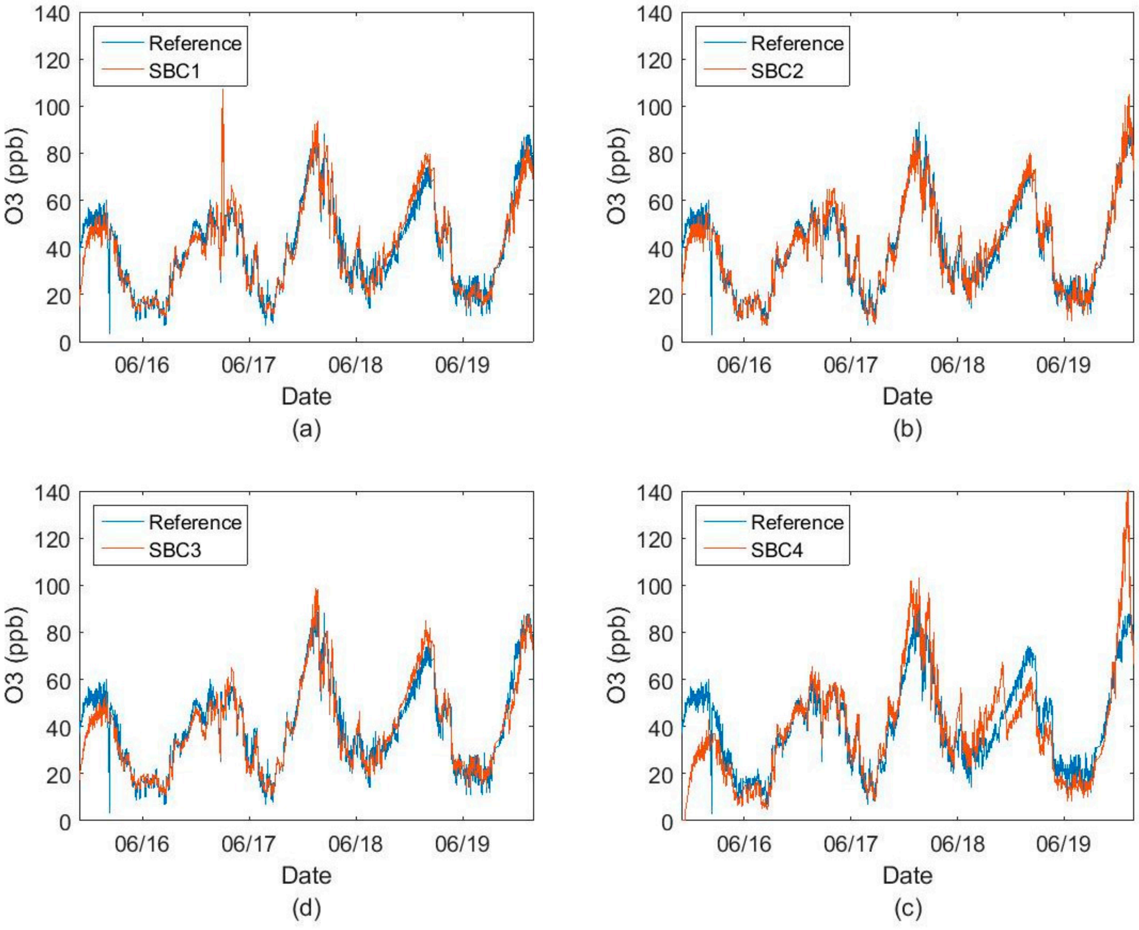

Figure S4. Time series of ozone measurements by SBC1 (**a**), SBC2 (**b**), SBC3 (**c**), and SBC4 (**d**) during validation data period with UPod data (red) and reference data (blue) shown in each plot.
